# Inflammatory Biomarkers and Sex Hormones Interact to Predict Ecologically-Assessed Cognitive Performance

**DOI:** 10.1093/geroni/igab046.3616

**Published:** 2021-12-17

**Authors:** Erik Knight, Erin Harrington, Martin Sliwinski, Jennifer Graham-Engeland, Jelena Pavlovic, Christopher Engeland

**Affiliations:** 1 University of Colorado Boulder, Boulder, Colorado, United States; 2 Pennsylvania State University, University Park, Pennsylvania, United States; 3 The Pennsylvania State University, University Park, Pennsylvania, United States; 4 Penn State University, University Park, Pennsylvania, United States; 5 Albert Einstein College of Medicine, Bronx, New York, United States

## Abstract

Inflammatory biomarkers and sex hormones have been investigated as independent risk and resilience factors for cognitive decline in older adults. Many sex hormones are anti-inflammatory and there is emerging evidence that sex hormones may buffer the risk for cognitive decline associated with higher inflammation. However, few studies have included concurrent examination of inflammation and sex hormones in studies of cognitive performance and cognitive aging. A diverse sample of older adults (N = 245; 65% female, 42% Black, 13% Hispanic; mean age = 76.8 years) had blood drawn before and after a two-week measurement burst that included three cognitive tests (6x per day) assessing working spatial memory, perceptual speed, and feature binding. Testosterone, estradiol, estrone, and six basal cytokine concentrations were quantified. Composite scores of basal inflammation were calculated. Multilevel modeling indicated that heightened inflammation related to poorer spatial working memory performance (B = 0.213, 95%CI[0.11, 0.414], p = .040). In addition, sex hormones moderated the association of cytokine concentration with perceptual speed (e.g., basal cytokines x testosterone: B = 0.13, [-0.24, -0.03], p = 0.013; similar effects evident for estrogens). Decomposition these interactions revealed that heightened inflammation predicted poorer performance, but only among individuals with lower sex-hormone concentrations. This study provides evidence of immune and hormonal-by-immune associations with performance in two cognitive domains in older adults. Examining the functional crosstalk between immune and sex hormone functioning will improve understanding of risk and resilience factors related to cognitive performance and help predict cognitive decline in older adults.

